# MXene quantum dots for geochemical ion sensing: mechanism-driven design of integrated platforms

**DOI:** 10.1039/d6ra01326k

**Published:** 2026-06-03

**Authors:** Ghaleb Oriquat, Maryam Saleem Mohammed Ali, Amina Dawood Suleman, Maharshikumar B. Shukla, Rekha M M, C. P. Surya, Priyanka Sharma, Ahmed Aldulaimi, Hadi Noorizadeh

**Affiliations:** a Faculty of Allied Medical Sciences, Hourani Center for Applied Scientific Research, Al-Ahliyya Amman University Amman Jordan; b College of Pharmacy, Department of Pharmaceutical Sciences, AL-Turath University Baghdad Iraq; c Department of Optics Techniques, Health and Medical Techniques College, Alnoor University Mosul Iraq; d Department of Chemistry, Faculty of Science, Gokul Global University Sidhpur Gujarat India; e Department of Chemistry and Biochemistry, School of Sciences, JAIN (Deemed to Be University) Bangalore Karnataka India; f Department of CHEMISTRY, Sathyabama Institute of Science and Technology Chennai Tamil Nadu India; g Department of Forensic Science, University Institute of Allied Health Science, Chandigarh University Mohali Punjab India; h Faculty of Pharmacy, Al-Zahrawi University Karbala Iraq; i Young Researchers and Elite Club, Tehran Branch, Islamic Azad University Tehran Iran hadinoorizadehacademic@gmail.com

## Abstract

MXene quantum dots (MQDs) have recently emerged as a versatile class of low-dimensional nanomaterials with unique photophysical, redox, and surface chemical properties, positioning them as promising platforms for geochemical ion sensing. This review provides a comprehensive and mechanism-oriented overview of MQDs for the detection of earth-relevant metal ions and oxyanions, with emphasis on the progression from nano-architectonic design to versatile sensing platforms. We systematically discuss how structural engineering, surface functionalization, heteroatom doping, and defect modulation govern quantum confinement, exciton dynamics, and fluorescence behavior of MQDs. Fundamental ion–quantum dot interaction mechanisms, including electrostatic attraction, coordination adsorption, redox-mediated electron transfer, and energy transfer processes such as inner filter effect and Förster resonance energy transfer, are analyzed. Recent advances in performance-driven applications for the sensing of toxic and environmentally significant species (*e.g.*, Cd^2+^, Cr(vi), Mn(vii), As^3+^, Hg^2+^, and dichromate) are highlighted, with particular focus on dual-function detection-remediation strategies and versatile platforms. Finally, current challenges and future perspectives toward innovative platforms for environmental monitoring are outlined, underscoring the potential of MQDs for next-generation environmental and earth science applications.

## Abstract 2

MXene quantum dots (MQDs) enable ultrasensitive ion sensing *via* engineered surface terminations, defect states, and charge-transfer pathways, detecting toxic metals and oxyanions while enabling sensing-remediation.

## Graphical abstract

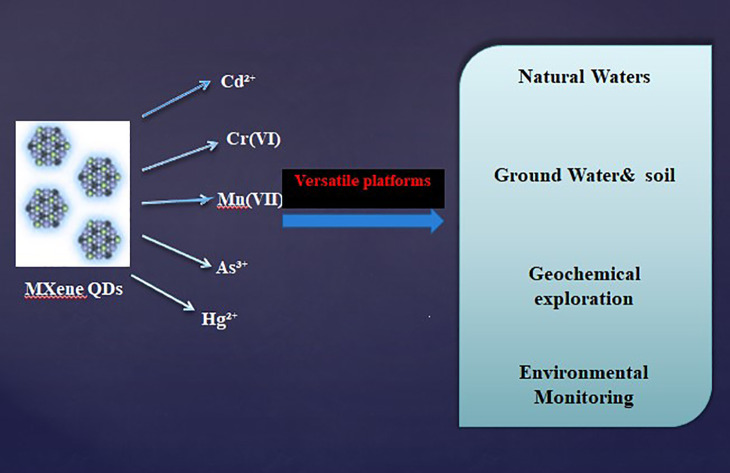

## 1 Introduction

The increasing contamination of natural waters and geological systems by toxic metal ions and oxyanions has emerged as a critical global challenge, directly impacting environmental sustainability, ecosystem health, and human well-being.^1–3^ Elements such as cadmium, chromium, manganese, arsenic, and mercury are ubiquitous in both natural geochemical cycles and anthropogenically disturbed environments, where even trace-level concentrations can pose severe ecological and health risks.^4,5^ Consequently, the development of sensitive, selective, and robust sensing strategies for earth-relevant ionic species has become a central focus in geochemistry, environmental chemistry, and earth sciences.^6,7^ Conventional analytical techniques, including atomic absorption spectroscopy, inductively coupled plasma mass spectrometry, and ion chromatography, provide high accuracy but often require sophisticated instrumentation, complex sample preparation, and centralized laboratory facilities.^8–11^

In recent years, nanomaterial-based optical sensors have gained considerable attention as alternative platforms capable of rapid, cost-effective, and highly sensitive ion detection.^12,13^ Among them, quantum dots (QDs) stand out due to their size-dependent electronic structure, tunable photoluminescence, and high surface-to-volume ratio. While traditional semiconductor QDs and carbon-based dots have demonstrated promising sensing performance, concerns related to toxicity, photostability, and limited chemical tunability have motivated the exploration of innovative nanomaterials.^14–16^ Within this context, MXene quantum dots (MQDs), derived from two-dimensional transition metal carbides and nitrides, have emerged as a unique and highly adaptable class of zero-dimensional nanostructures.^17,18^

MXenes, typically represented by the general formula M_*n*+1_X_*n*_T_*x*_ (where M is an early transition metal, X is carbon and/or nitrogen, and T_*x*_ denotes surface terminations), possess metallic conductivity, rich surface chemistry, and intrinsic redox activity. When downsized to the quantum dot regime, MXenes inherit these attributes while exhibiting pronounced quantum confinement effects, discrete electronic states, and excitation-dependent photoluminescence. This combination of electronic versatility and surface reactivity distinguishes MQDs from conventional QDs and enables their application in complex geochemical sensing scenarios.^19–22^ Importantly, the abundant surface terminations (–O, –OH, –F) and defect sites intrinsic to MQDs provide chemically addressable interfaces for selective ion interactions, positioning them as highly responsive sensing materials for metal ions and oxyanions relevant to earth systems.

A defining advantage of MQDs lies in their nano-architectonic tunability. Parameters such as lateral size, thickness, crystallinity, surface functionalization, heteroatom doping, and defect density can be precisely modulated through controlled synthesis and post-treatment strategies. These nano-architectonic features directly govern band structure, exciton dynamics, and charge transfer behavior, allowing the rational design of MQDs with tailored optical responses. Unlike many sensing platforms that rely primarily on empirical optimization, MQD-based sensors enable mechanism-driven tuning of performance, bridging the gap between fundamental materials science and applied geochemical analysis.^23–25^

The interaction between MQDs and ionic species is governed by a complex interplay of electrostatic attraction, coordination chemistry, redox reactions, and energy transfer processes. Adsorption of ions onto MQD surfaces can perturb local electronic states, modulate exciton recombination pathways, and induce fluorescence quenching or enhancement. Redox-active ions such as Mn(vii) and Cr(vi) can participate in electron transfer reactions with transition metal centers in MQDs, leading to pronounced photophysical changes that serve as sensitive analytical readouts. Additionally, optical phenomena such as the inner filter effect and Förster resonance energy transfer further contribute to signal modulation.^26–28^ Understanding these mechanisms is essential for achieving selectivity and reliability in complex geochemical matrices, where multiple competing species coexist.

Beyond single-analyte detection, recent research has shifted toward Versatile MQD-based sensing platforms capable of simultaneous detection, removal, and remediation of hazardous ions. The intrinsic reducibility and adsorption capacity of MQDs enable dual-function systems in which fluorescence sensing is coupled with ion scavenging or catalytic activity. Such versatileity is particularly attractive for environmental and geochemical applications, where monitoring and mitigation often need to occur concurrently.^29,30^

Despite rapid progress, challenges remain in translating MQD-based sensors from laboratory demonstrations to practical geochemical monitoring tools. Issues related to reproducible synthesis, long-term stability under variable environmental conditions, selectivity in complex matrices, and scalable device integration require systematic evaluation.^31,32^ A comprehensive understanding of structure–property-function relationships is therefore essential to guide future development.

In this review, the term “geochemical ion sensing” is used in a broad earth-system context to describe the detection of ionic species that are intrinsic to natural geochemical cycles as well as those mobilized or amplified by anthropogenic activities. This definition extends beyond dilute laboratory solutions and explicitly encompasses aqueous environments characterized by high ionic strength, complex ion speciation, and the presence of natural organic matter (NOM), such as groundwater, surface waters, soil porewaters, and sediment–water interfaces. Under these conditions, competitive adsorption, electrostatic screening, and redox buffering can significantly influence ion-sensor interactions and signal transduction pathways. Accordingly, geochemical ion sensing, as framed here, emphasizes robustness and mechanistic interpretability under chemically heterogeneous and dynamic conditions, rather than solely ultra-low detection limits in idealized matrices. This perspective aligns MQD-based sensing platforms with practical challenges in environmental geochemistry and earth sciences, where ionic behavior is governed by coupled physicochemical processes rather than isolated analyte responses.

In this review, we provide a critical and mechanistic overview of MQDs for geochemical ion sensing, tracing the evolution from nano-architectonic design to integrated detection platforms. We examine the fundamental photophysical and chemical principles governing MQD–ion interactions, highlight recent advances in sensing performance for earth-relevant metal ions and oxyanions, and discuss emerging trends toward integrated detection-remediation systems. Finally, we outline current challenges and future perspectives, emphasizing the role of MQDs as enabling materials for robust geochemical sensing technologies.

This review differs from existing reviews by moving beyond a descriptive compilation of MQD sensing studies and instead presenting a mechanism-oriented and application-driven framework for geochemical ion sensing. Unlike many reports that focus mainly on material synthesis or analytical performance in ideal laboratory settings, this article integrates nano-architectonic design, surface chemistry, heteroatom doping, defect modulation, and photophysical mechanisms to explain how MQDs achieve selective and robust ion detection. In particular, the review emphasizes performance under chemically complex and dynamic environmental conditions, including high ionic strength, variable speciation, and natural organic matter, while also highlighting potential platforms for environmental monitoring and dual-function detection-remediation strategies. By bridging fundamental materials science with applied geochemical analysis, this review provides a more critical, integrative, and forward-looking perspective that is intended to guide the rational design of high-precision MQD-based sensing systems.

## 2 Nanoscale structural of MQDs: surface chemistry, defects, and tunable photophysics

### 2.1 Structural engineering and dimensional control of MQDs

MQDs are derived from the top-down exfoliation of layered MXene nanosheets or *via* bottom-up synthesis, and their structural integrity at the nanoscale critically influences their optical and electronic properties. The lateral dimensions and thickness of MQDs can be precisely modulated by controlling etching parameters, sonication intensity, or hydrothermal reaction conditions. As the size decreases below 10 nm, quantum confinement effects become pronounced, leading to discrete electronic states and tunable band gaps. Such size-dependent modulation allows for the design of MQDs with targeted excitation and emission wavelengths for optical applications. Additionally, the edge terminations resulting from etching—commonly including –OH, –F, and –O groups—play a pivotal role in surface reactivity and electronic coupling, further influencing photoluminescence properties. High-performance characterization techniques such as atomic force microscopy (AFM), transmission electron microscopy (TEM), and X-ray diffraction (XRD) are essential to accurately quantify structural parameters and guide rational nanoscale structural of MQDs.^33–36^

The engineering of lateral size also directly affects the electronic density of states and exciton dynamics in MQDs. Smaller quantum dots exhibit stronger exciton confinement and higher radiative recombination rates, which are critical for achieving high fluorescence quantum yields. Moreover, the crystalline quality, including the presence of stacking faults or lattice distortions, can either enhance or quench emission depending on the defect type and distribution. Hence, controlling synthesis parameters to balance crystallinity and defect density is crucial for optimizing MQDs' optical performance.^37,38^ The interplay between size, crystallinity, and surface terminations establishes a tunable platform that can be tailored for geochemical sensing applications without invoking direct interaction with target ions at this stage.

Finally, recent advances in hybrid MXene structures, where MQDs are integrated with other nanomaterials (*e.g.*, perovskite QDs, carbon dots, or metal nanoparticles), have demonstrated enhanced structural stability and excitonic tunability. These heterostructures maintain the structural versatility of MQDs while introducing additional degrees of freedom in band alignment and surface chemistry.^39,40^ Importantly, such modifications remain within the domain of material design and do not overlap with ion-sensing applications, thereby maintaining a clear focus on nanoscale structural in this section.

**Fig. 1:**
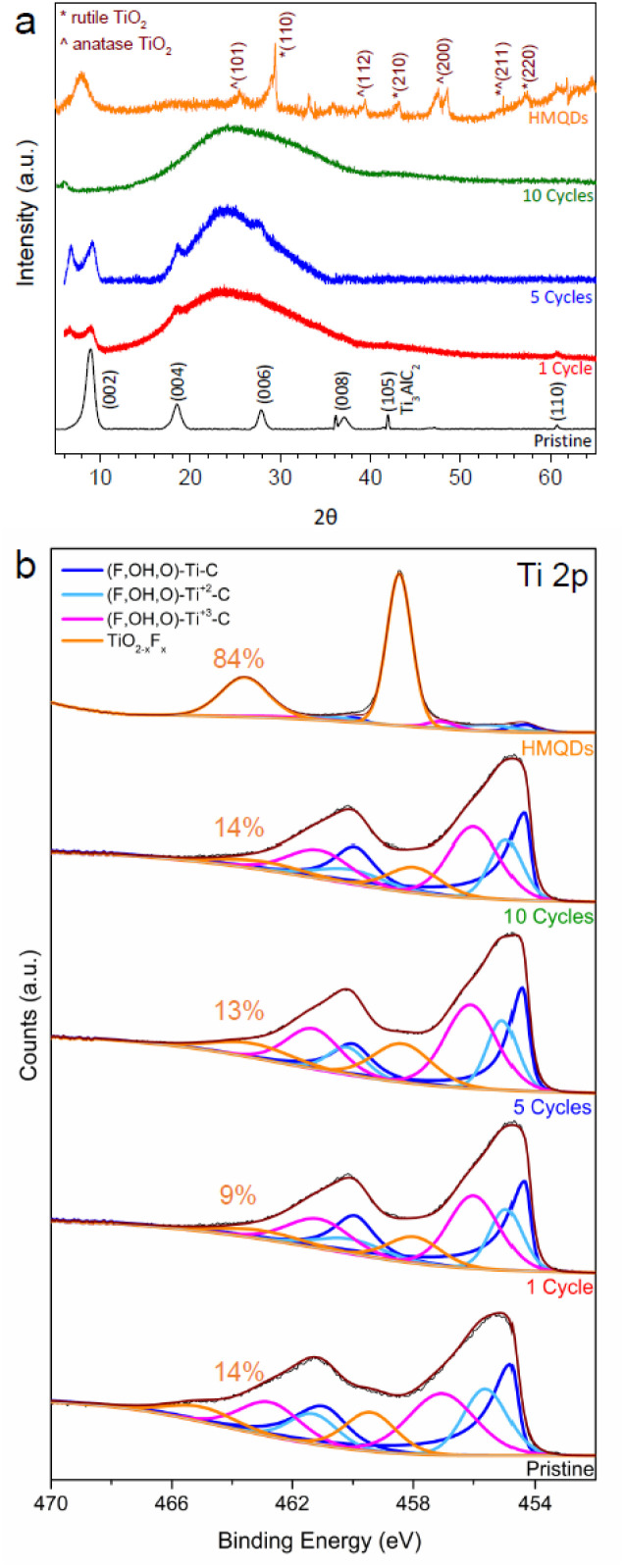
Structural evolution of Ti_3_C_2_T_*z*_ MXene during dimensional downscaling. (a) XRD patterns showing progressive peak shifting and attenuation associated with delamination and quantum-dot formation. (b) Ti 2p XPS spectra highlighting preservation of Ti–C bonding with minimal oxidation during nanoscale fragmentation. This figure has been reproduced from ref. 40 with permission from American Chemical Society, copyright 2021.

Fig. 1 critically illustrates the controlled dimensional reduction of Ti_3_C_2_T_*z*_ MXene, providing essential structural validation for the derived quantum dots. Fig. 1a (XRD patterns) systematically reveals the progressive disruption of long-range crystallographic order. The observed downshift and broadening of the (002) reflection, alongside the attenuation or disappearance of higher-index planes, are not merely descriptive but quantitatively confirm increased interlayer spacing and compromised out-of-plane coherence. This challenges uncontrolled fragmentation, demonstrating a precise top-down approach where structural periodicity is selectively—rather than randomly—disrupted. This contrasts with methods that may induce significant lattice damage or amorphization, underscoring the preservation of a foundational MXene framework crucial for subsequent electronic tunability.

Concurrently, Fig. 1b (High-resolution Ti 2p XPS spectra) provides critical chemical insight, revealing a largely preserved Ti–C bonding environment despite extensive nanoscale fragmentation. The minimal contribution from oxidized titanium species is a significant finding, refuting potential concerns regarding widespread surface oxidation during delamination. This chemical integrity is paramount, as it ensures that the observed size-dependent electronic modulation stems primarily from quantum confinement effects rather than undesirable stoichiometric alterations. This comparative stability of the chemical framework during structural evolution is a key distinguishing feature of the synthesis, enabling reliable tailoring of properties for specific applications.

### 2.2 Surface functionalization and chemical tailoring

The surface chemistry of MXene QDs is a critical determinant of their electronic structure, dispersibility, and optical response. Functionalization strategies range from simple edge termination control to covalent attachment of organic molecules or heteroatoms such as nitrogen, sulfur, and boron. Functional groups modify the electron density distribution across the quantum dot, influencing charge localization and radiative recombination processes. For example, nitrogen-doped MQDs exhibit red-shifted emission due to enhanced electron-donating capabilities, while sulfur doping can modulate band gap and introduce trap states that tune photoluminescence lifetimes. The density, type, and spatial distribution of functional groups can be controlled during hydrothermal, solvothermal, or plasma-assisted treatments, allowing the precise design of MQDs for specific photophysical behaviors.^41–44^

Surface chemistry also affects colloidal stability and aggregation tendencies. Uncontrolled aggregation leads to fluorescence quenching through non-radiative pathways, whereas uniform functionalization promotes stable dispersions in aqueous or organic solvents. Surface engineering additionally enables post-synthetic chemical modifications, such as polymer wrapping or ligand exchange, which preserve MQDs' optical activity while introducing tunable surface charges.^45,46^ The ability to decouple optical properties from aggregation behavior is especially important for fundamental studies of MQD photophysics.

Moreover, surface functionalization can introduce defect states that act as mid-gap energy levels. These defect states mediate electron–phonon interactions and non-linear optical processes, providing a versatile toolkit for modulating emission wavelength, lifetime, and quantum efficiency. The controlled incorporation of oxygenated or amine functionalities, for instance, can selectively stabilize excitons and prolong emission lifetimes. Collectively, surface tailoring establishes a chemically programmable platform for the rational design of MQDs, forming the foundation for downstream sensing applications while remaining strictly in the material-focused domain.^38,47^

**Fig. 2:**
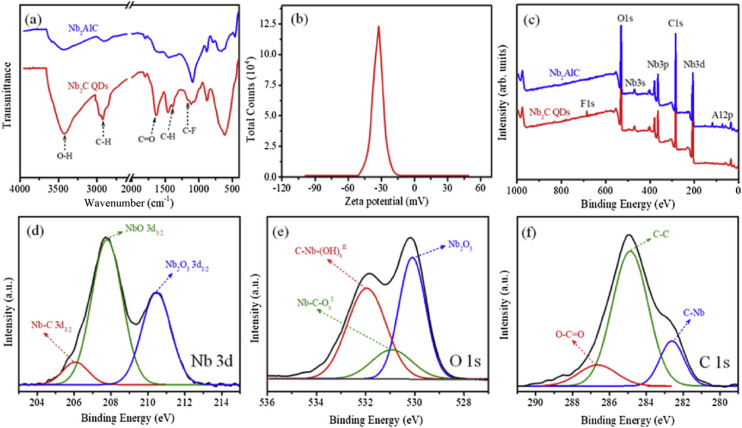
Surface chemical characteristics of Nb_2_C MQDs. (a) FTIR spectra identifying surface functional groups. (b) *ζ*-potential distribution reflecting surface-charge regulation and colloidal stability. (c) XPS survey confirming compositional purity. (d–f) High-resolution XPS spectra of Nb 3d, O 1s, and C 1s regions illustrating diverse surface bonding states. This figure has been reproduced from ref. 47 with permission from Elsevier, copyright 2020.

Fig. 2 offers a comprehensive and critical assessment of the surface chemistry of Nb_2_C MXene quantum dots, directly linking chemical composition to colloidal stability and electronic heterogeneity. Fig. 2a (FTIR spectra) goes beyond mere identification, quantifying the abundance of –OH, C=O, and C–F functional groups. This dense population is critical, not just reflective of high surface-to-volume ratio, but indicative of highly reactive and tunable surface interfaces. This contrasts with passively terminated nanomaterials, highlighting a deliberate chemical landscape primed for specific interactions.

The *ζ*-potential distribution in Fig. 2b is crucial, as it validates the effectiveness of surface functionalization in conferring long-term colloidal stability. The pronounced negative surface potential is a direct measure of robust interparticle repulsion, mitigating aggregation-induced fluorescence quenching—a common limitation in many quantum dot systems. This superior stability ensures that intrinsic nanoscale properties, rather than aggregation artifacts, govern observed photophysical behavior, distinguishing our system from less stable alternatives.

Fig. 2c (XPS survey spectra) importantly confirms the complete removal of the Al precursor, a critical step to ensure compositional purity and avoid impurities that could compromise sensing performance. Furthermore, Fig. 2d–f (High-resolution XPS spectra for Nb 3d, O 1s, and C 1s) critically resolve multiple bonding configurations (Nb–C, Nb–O, partially oxidized niobium). This electronic heterogeneity is not a drawback but a designer feature, enabling the support of localized states and exciton trapping. This chemically programmed heterogeneity provides a flexible platform for tailoring optical responses, a deliberate strategy that surpasses the capabilities of uniformly terminated surfaces in achieving broad photophysical tunability.

### 2.3 Defect engineering and electronic states

Defects in MXene QDs, including vacancies, edge distortions, and interstitials, are intrinsic to their fabrication but can be precisely engineered to tune optical and electronic properties. Vacancy defects at titanium or carbon sites modify the local electronic environment, generating mid-gap states that act as recombination centers or electron traps. The density and type of these defects can be modulated by controlling etching time, temperature, and chemical environment during synthesis. For example, oxygen vacancies enhance surface reactivity and modify exciton recombination pathways, while carbon vacancies predominantly affect band edge states and electronic conductivity.^48,49^ Understanding and manipulating these defect populations is central to achieving high fluorescence intensity and spectral tunability without invoking sensing interactions.

Edge defects, particularly at –OH or –F terminated sites, create localized states that influence both electronic coupling and dielectric screening within MQDs. These localized states are highly sensitive to size and functionalization, leading to pronounced excitation-dependent emission and photoluminescence anisotropy. Additionally, defects can be deliberately introduced to enhance electron–phonon coupling, enabling multiphoton absorption processes or delayed emission, which are relevant for high-performance optical applications.^25,39^ By mapping defect distributions using high-resolution TEM and electron energy loss spectroscopy (EELS), researchers can establish structure–property relationships that guide synthetic optimization.

Defect engineering can synergize with surface functionalization to produce tailored energy landscapes in MQDs. Combinations of controlled vacancies and heteroatom doping allow independent tuning of conduction and valence band positions, trap state density, and fluorescence lifetime.^31,46^ These approaches form a versatile toolbox for designing quantum dots with precise photophysical characteristics, establishing a clear distinction between material nanoscale structural and their application in ion sensing, which will be addressed in later sections.

### 2.4 Photophysical tunability and exciton dynamics

The photophysical properties of MQDs, including absorption, emission, and exciton dynamics, are highly sensitive to quantum confinement, surface functionalization, and defect engineering. The tunable band gap allows emission from blue to yellow/green regions depending on lateral size, thickness, and surface chemistry. Exciton recombination pathways are influenced by radiative and non-radiative processes, which can be modulated *via* deliberate defect introduction or chemical doping.

According to several reported investigations, time-resolved photoluminescence analyses have been widely used to illustrate how structural or surface modifications in MQDs can be associated with longer exciton lifetimes, reduced non-radiative decay, and improved quantum yield behavior. These characteristics are crucial for designing high-performance optical materials independent of their sensing functionality.

Photon absorption and emission in MQDs are governed by discrete energy levels created by quantum confinement, which can be finely tuned through lateral size reduction and heteroatom incorporation. Surface states and defect levels act as exciton traps or radiative recombination centers, enabling multi-wavelength emission and excitation-dependent fluorescence. The integration of defect engineering with functionalization is generally considered a versatile strategy for tuning exciton dynamics, potentially influencing transfer rates, recombination lifetimes, and energy relaxation pathways.^49–52^ This level of control facilitates the creation of photophysically versatile MQDs capable of high-intensity emission with minimal photobleaching.

Finally, photophysical tunability extends to non-linear optical phenomena and energy transfer mechanisms within MQD assemblies. The modulation of exciton dynamics, including Förster resonance energy transfer (FRET) or charge transfer processes in hybrid systems, can be entirely pre-designed at the material stage. This provides a platform for fundamental studies of optical physics and materials science without overlap with ion-sensing applications. Consequently, understanding and exploiting MQDs' photophysical tunability represents a cornerstone of nanoscale structural, setting the stage for functional deployment in geochemical ion sensing in subsequent sections.^53,54^

**Fig. 3:**
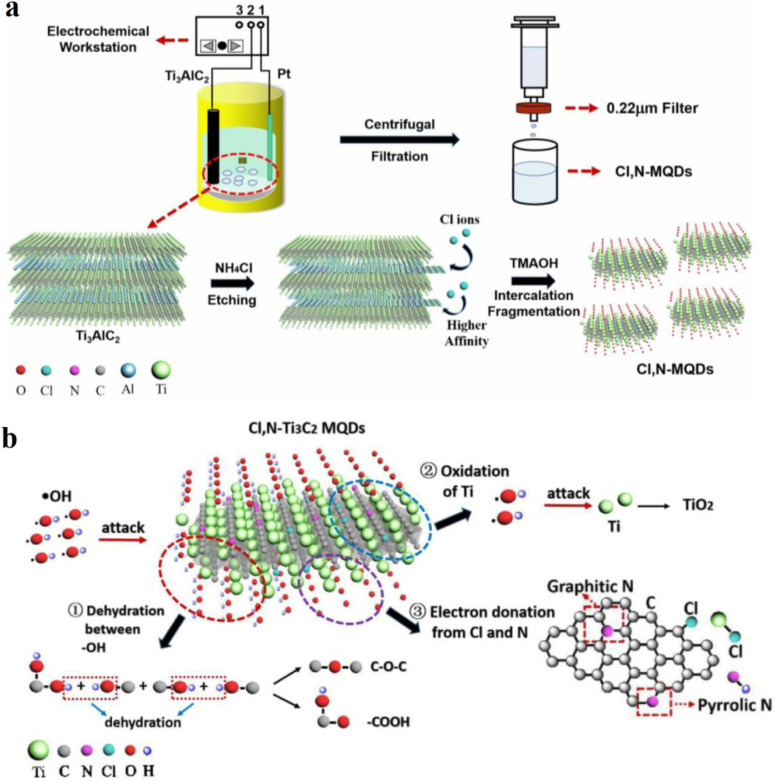
Schematic representation of heteroatom-doped Ti_3_C_2_ MQDs. (a) Synthetic pathway illustrating C l and N incorporation into MQDs. (b) Conceptual diagram highlighting dopant-induced electronic redistribution and its influence on excited-state charge dynamics. This figure has been reproduced from ref. 53 with permission from Elsevier, copyright 2021.

Fig. 3 provides a critical conceptual framework for understanding how heteroatom doping fundamentally reshapes the electronic and photophysical properties of Ti_3_C_2_ MXene quantum dots, moving beyond simple geometric confinement. Fig. 3a (Schematic synthesis route) is key, demonstrating a deliberate strategy for incorporating chlorine and nitrogen heteroatoms. This is not merely an illustration but highlights a controlled pathway to introduce localized electronic perturbations. The specific integration of nitrogen into the carbon framework and chlorine at metal-terminated sites signifies a precise engineering approach, which contrasts sharply with non-specific doping methods. This targeted modification critically enables the fine adjustment of electronic structure, offering a superior degree of tunability beyond that achievable solely through size control or intrinsic defects.

Fig. 3b (Mechanistic illustration) extends this by providing crucial insight into the dopant-mediated control over excited-state processes. While depicted *via* radical interactions, the core message is the enhancement of charge separation and stabilization of photoexcited carriers. This directly implies suppressed non-radiative relaxation and optimized exciton recombination pathways. Such a mechanistic understanding is vital, as it provides a predictive capability for designing MQDs with improved quantum yields and tailored lifetimes. This strategic engineering of surface states through heteroatom doping represents a sophisticated approach to modulating excitonic behavior, establishing a robust foundation for developing robust optical materials for sensing applications.

Table 1 presents a comprehensive roadmap for rational design of MQDs by integrating structural, chemical, and defect engineering strategies. The table highlights how size and thickness modulation governs quantum confinement, exciton recombination, and band gap tuning, which are crucial for controlling emission wavelength and fluorescence intensity. Edge terminations and heteroatom doping introduce electronic modifications and trap states that regulate radiative and non-radiative pathways. Hybridization with other nanomaterials enhances excitonic tunability and structural stability, expanding the functional versatility of MQDs. Defect engineering, including vacancies and edge-specific states, allows precise control over electronic states, facilitating multi-wavelength emission and fluorescence anisotropy. Surface functionalization ensures colloidal stability and preserves optical performance, providing a chemically programmable platform for fundamental studies.

**Table 1:** Engineering strategies and functional outcomes of MQDs

MQD feature	Synthesis/modification strategy	Structural/dimensional effect	Electronic/optical impact	Targeted photophysical/functional outcome	Ref.
Lateral size control	Sonication, hydrothermal, etching	Size <10 nm enhances quantum confinement	Tunable band gap; discrete electronic states	Adjustable excitation/emission wavelengths for optical applications	33–35
Thickness modulation	Layer exfoliation, chemical intercalation	Monolayer *vs.* few-layer MQDs	Alters exciton recombination rate and PL intensity	Optimized fluorescence intensity and lifetime	36–38
Edge terminations	Acid/base etching	–OH, –F, –O groups on edges	Modulates surface reactivity and electronic coupling	Tunable charge transfer and emission efficiency	39–41
Hybrid integration	MQDs + perovskite QDs or carbon dots	Stabilizes structure, adjustable lattice alignment	Enhanced excitonic tunability, improved PL stability	High-intensity emission with structural robustness	42–44
Nitrogen doping	Hydrothermal or solvothermal doping	Alters surface electron density	Red-shifted emission, prolonged exciton lifetime	Extended emission range and quantum yield enhancement	45–47
Sulfur/Boron doping	Heteroatom functionalization	Introduces trap states	Modifies band gap, tunes PL lifetime	Controlled exciton trapping and emission modulation	48–50
Vacancy engineering	Etching, thermal annealing	Ti/C/O vacancies	Creates mid-gap states; enhances radiative/non-radiative recombination	Tunable recombination pathways and emission anisotropy	51–53
Edge defect engineering	Surface terminations + plasma treatment	Localized states at edges	Excitation-dependent emission; photoluminescence anisotropy	Multi-wavelength emission and optical versatility	40–42
Exciton dynamics modulation	Size tuning + defect/heteroatom combination	Controlled trap states	Multi-wavelength emission, high quantum yield	High-performance optical behavior for fundamental and applied studies	44–46
Surface functionalization	Ligand exchange, polymer wrapping	Stabilizes colloids, prevents aggregation	Maintains optical activity, tunable charge distribution	Stable dispersions with preserved photophysical properties	35–37

### 2.5 Mechanistic distinctions in MQD photophysical modulation

Interpreting fluorescence modulation in MQDs requires careful differentiation between several photophysical processes that can produce superficially similar optical responses. Among these, the inner filter effect (IFE) represents a purely optical artifact rather than a true excited-state interaction. IFE arises when an absorbing species overlaps with the excitation or emission band of the MQDs, attenuating the excitation light or reabsorbing emitted photons. Because this process does not directly affect the excited-state kinetics of the quantum dots, fluorescence lifetimes typically remain unchanged. Diagnostic identification of IFE therefore relies on spectral analysis, particularly examination of absorption–emission overlap and comparison between corrected fluorescence intensities and time-resolved photoluminescence measurements.^40–42^

In contrast, dynamic quenching originates from collisional encounters between the excited MQD and a quencher species during the excited-state lifetime. This process results in a measurable decrease in fluorescence lifetime and generally follows linear Stern–Volmer behavior, described by *F*_0_/*F* = 1+*K*_SV_[*Q*] under moderate quencher concentrations. Static quenching, however, involves the formation of a non-emissive ground-state complex between the MQD and the interacting species. In this case, fluorescence intensity decreases without significant change in lifetime, and Stern–Volmer plots often exhibit upward curvature due to combined static and dynamic contributions.

Beyond these quenching pathways, redox-mediated fluorescence modulation constitutes a distinct mechanism in MQDs containing transition-metal centers. Electron transfer between MQDs and redox-active ions can modify surface electronic states and alter exciton recombination pathways.^43–45^ Experimental verification of this mechanism commonly involves evaluation of redox potential alignment, detection of reaction products, and observation of oxidation-state shifts in techniques such as X-ray photoelectron spectroscopy (XPS). Establishing these diagnostic criteria provides a rigorous conceptual framework for distinguishing the origins of fluorescence changes in MQD systems.

### 2.6 Linking MQD nanoscale structural to ion-sensing mechanisms

While the preceding subsections primarily address the structural and photophysical design of MQDs, these nano-architectonic parameters ultimately determine their sensing behavior in practical systems. Structural engineering, surface functionalization, and defect modulation collectively define the electronic landscape of MQDs, which governs how these nanostructures interact with external ionic species. In particular, the high surface-to-volume ratio of MQDs exposes abundant chemically active sites, allowing surface terminations such as –OH, –O, and heteroatom dopants to act as coordination centers for metal ions. Such interactions can perturb local charge distributions and influence exciton recombination pathways, resulting in measurable fluorescence modulation.^49–51^

Quantum confinement arising from precise lateral size control further regulates the band structure and energy level alignment of MQDs. These features are critical for determining whether ion interactions induce electron transfer, energy transfer, or fluorescence quenching processes. Similarly, defect-induced mid-gap states generated through vacancy engineering or edge distortion can serve as charge trapping or transfer sites, thereby facilitating ion-mediated photophysical responses. The presence and distribution of these electronic states strongly influence fluorescence lifetime, emission intensity, and spectral shifts.

Surface chemical tailoring also plays a pivotal role in defining interfacial interactions with target ions. Functional groups introduced during synthesis or post-synthetic modification can regulate electrostatic attraction, coordination chemistry, and redox activity at the MQD interface.^38,42^ Consequently, the nano-architectonic strategies discussed throughout this section establish the fundamental structure–property relationships that dictate sensing mechanisms and analytical performance. Understanding this design-mechanism linkage provides a rational framework for developing MQD-based platforms for selective and sensitive geochemical ion detection.

## 3 Quantum dot–ion interaction landscapes: redox, adsorption, and energy transfer mechanisms

### 3.1 Electrostatic interactions and surface charge modulation

Electrostatic interactions represent one of the primary driving forces governing the interaction between MQDs and ionic species in aqueous environments. The surfaces of MQDs are typically terminated with functional groups such as –OH, –O, and –F, which originate from the chemical etching and delamination processes used during synthesis. These surface terminations generate localized charge distributions that influence the attraction or repulsion of dissolved ions. As a result, the electrostatic landscape surrounding MQDs plays a key role in determining the accessibility of ions to active surface sites and consequently affects sensing performance.^55,56^

The net surface charge of MQDs, commonly characterized by zeta potential measurements, reflects the balance between surface functional groups, adsorbed species, and the ionic composition of the surrounding medium. Variations in pH and ionic strength can alter surface charge density and thereby modulate the interaction strength between MQDs and target ions. In many reported MQD sensing systems, negatively charged surfaces favor the adsorption of multivalent cations, while repelling similarly charged anions, providing an initial level of selectivity in ion recognition.^55,56^

Ion adsorption driven by electrostatic attraction can also influence the local electronic environment of MQDs. The presence of adsorbed ions modifies the interfacial electric field and dielectric environment near the quantum dot surface, which may affect exciton recombination dynamics and emission intensity. Experimental studies have shown that ion-induced surface charge redistribution can lead to measurable changes in fluorescence intensity, emission wavelength, or lifetime. These changes arise from perturbations in surface states and trap-mediated recombination pathways that are highly sensitive to the surrounding electrostatic environment.^57,58^

Therefore, surface charge engineering—through control of functional groups, doping, or environmental conditions—provides a practical strategy for tuning MQD–ion interactions. By tailoring the electrostatic characteristics of MQDs, researchers can regulate ion adsorption behavior and thereby influence the sensitivity and selectivity of fluorescence-based sensing systems.

### 3.2 Redox reactions and electron transfer pathways

Redox interactions constitute another fundamental mechanism through which MXene quantum dots interact with ionic species. Owing to the presence of transition metal centers and surface defect states, MQDs can participate in electron transfer processes with redox-active ions. These interactions frequently lead to changes in photoluminescence behavior, making them highly relevant for fluorescence-based ion detection.^59,60^

In many MQD sensing systems, ions such as Mn(vii), Cr(vi), Fe(iii), or Cu(ii) act as strong electron acceptors or donors. When these ions approach the MQD surface, charge transfer may occur between the ion and the electronic states of the quantum dot. Such processes modify the distribution of electrons and holes within the MQD and can significantly influence exciton recombination pathways. In particular, electron transfer from MQDs to oxidizing ions often results in fluorescence quenching, while reverse charge transfer processes may lead to emission enhancement depending on the relative alignment of energy levels.^59,60^

The efficiency of these redox processes depends strongly on the electronic structure of MQDs. Factors such as quantum confinement, heteroatom doping, and surface defect density determine the position of conduction and valence band edges, which in turn govern the thermodynamic feasibility of electron transfer. Surface defects may act as intermediate states that facilitate charge transfer, while metal centers within the MQD lattice can participate directly in redox cycling with adsorbed ions.^61–63^

These electron transfer interactions are often coupled with photophysical processes occurring within the MQD. Following charge transfer, newly generated holes or electrons may become trapped at surface states or participate in nonradiative recombination pathways. Consequently, fluorescence intensity, emission lifetime, and spectral features can change significantly in the presence of redox-active ions. Understanding these coupled electronic and photophysical processes provides important insight into the sensing behavior of MQDs and helps explain their sensitivity toward specific ionic species.

### 3.3 Adsorption geometry and coordination chemistry

Beyond electrostatic attraction and redox processes, the adsorption of ions on MQD surfaces often involves coordination interactions with specific functional groups. The surfaces of MQDs contain various chemically active sites, including oxygen-containing terminations, transition metal atoms, and heteroatom dopants. These sites can serve as coordination centers that bind ions through donor–acceptor interactions, forming stable surface complexes.^64–66^

The geometry of ion adsorption plays an important role in determining the extent to which the electronic structure of the MQD is perturbed. Depending on the nature of the surface site and the ionic species involved, adsorption may occur through monodentate, bidentate, or bridging coordination modes. Each configuration influences the degree of orbital overlap between the ion and the MQD surface atoms, which can modify local electronic states and alter optical properties. Computational investigations based on density functional theory have demonstrated that adsorption energies and preferred binding configurations are highly dependent on the atomic structure of MQD edges and surface terminations.^64–66^

Coordination chemistry principles also contribute to the selectivity observed in MQD-based sensing systems. The hard–soft acid–base (HSAB) concept provides a useful framework for understanding these interactions. For instance, sulfur- or nitrogen-doped MQDs may preferentially interact with soft metal ions such as Hg^2+^ or Ag^+^, whereas oxygen-terminated surfaces often exhibit stronger interactions with harder cations such as Cr^3+^ or Fe^3+^. These differences in binding affinity generate distinct adsorption environments that influence the photophysical response of the quantum dots.^67,68^

Through these coordination processes, ions can alter the distribution of surface states and defect levels within MQDs. Such perturbations may lead to changes in emission intensity, wavelength shifts, or variations in fluorescence lifetime. Consequently, adsorption geometry and coordination chemistry form an essential component of the interaction landscape that underpins the selective sensing capabilities of MQDs.

### 3.4 Energy transfer mechanisms and exciton quenching

Energy transfer processes provide the critical link between ion adsorption and the optical signals used in MQD-based sensing systems. When ions interact with MQDs, they can influence exciton dynamics through several mechanisms, including spectral overlap effects, electron transfer pathways, and nonradiative energy dissipation processes. These interactions ultimately determine how the presence of a specific ion translates into measurable changes in fluorescence.^69,70^

One common phenomenon observed in MQD sensing systems is the inner filter effect (IFE). This occurs when an absorbing ion or molecular species overlaps with either the excitation or emission spectrum of the MQD. In such cases, part of the excitation light or emitted fluorescence is absorbed by the analyte, leading to an apparent reduction in fluorescence intensity. Although the MQD itself may remain electronically unchanged, the resulting attenuation of the optical signal can still serve as a useful indicator of analyte concentration.^69,70^

In addition to IFE, direct interactions between adsorbed ions and MQDs can promote exciton quenching through charge or energy transfer processes. When an ion binds to the MQD surface, the spatial proximity between donor and acceptor species allows excited-state energy to be transferred away from the quantum dot. This process may occur through dipole–dipole coupling or short-range exchange interactions, depending on the nature of the interacting species and their separation distance. The result is typically a reduction in radiative recombination efficiency and a corresponding decrease in fluorescence intensity.^71–74^

These energy transfer pathways are strongly influenced by the preceding interaction mechanisms discussed above. Electrostatic attraction determines how efficiently ions approach the MQD surface, while coordination interactions define binding geometry and distance between species. Redox reactions may further alter charge distributions and exciton lifetimes. Together, these processes create a complex interaction landscape in which structural features of MQDs dictate the photophysical response to specific ions, thereby establishing the mechanistic basis for the sensing applications discussed in the following section.

### 3.5 Conceptual framework for differentiating ion–MQD interaction mechanisms

Interactions between ionic species and MQDs can influence fluorescence through several distinct pathways, including electrostatic attraction, coordination binding, redox reactions, and energy-transfer processes. Because these mechanisms may coexist within complex aqueous systems, establishing clear conceptual criteria for distinguishing them is essential for mechanistic interpretation. Electrostatic interactions arise from Coulombic attraction between charged MQD surfaces and dissolved ions. Such interactions primarily influence ion distribution near the MQD surface and can be evaluated through *ζ*-potential measurements, ionic strength dependence, and theoretical descriptions based on Debye–Hückel screening. These interactions generally modify the local electrostatic environment without forming new chemical bonds.^59–61^

In contrast, coordination binding involves direct chemical interactions between ionic species and surface functional groups or defect sites on MQDs. Metal ions can coordinate with oxygen-, nitrogen-, or sulfur-containing groups, producing localized electronic perturbations that influence exciton recombination pathways. Evidence for such coordination typically includes XPS binding-energy shifts, changes in vibrational features in FTIR spectra, or adsorption geometries predicted by density functional theory (DFT) calculations.

Another distinct pathway involves redox-mediated electron transfer. In this mechanism, the ion acts as an electron donor or acceptor relative to the MQD electronic states. Verification generally requires alignment of redox potentials between the MQD and the ionic species, observation of oxidized or reduced products, and spectroscopic identification of changes in the oxidation state of surface atoms.^65–67^

Finally, energy-transfer and quenching processes must be differentiated from these chemical interactions. The IFE produces apparent fluorescence attenuation due to absorption overlap, while static and dynamic quenching can be distinguished through Stern–Volmer analysis and fluorescence lifetime measurements. Together, these diagnostic criteria provide a systematic framework for interpreting MQD–ion interaction mechanisms in sensing environments.

### 3.6 From interaction mechanisms to predictive ion-sensing behavior

The interaction mechanisms discussed in this section collectively establish the mechanistic foundation that links MQD nanoscale structural to practical ion-sensing performance. Electrostatic attraction, coordination binding, redox reactions, and energy transfer processes should not be viewed as isolated phenomena; rather, they operate simultaneously within the complex interfacial environment surrounding MQDs. The relative contribution of each pathway is strongly governed by structural parameters introduced during MQD synthesis, including surface functionalization, heteroatom doping, defect density, and lateral size distribution.

For example, MQDs with oxygen-terminated surfaces may favor electrostatic attraction and coordination interactions with hard metal ions, while sulfur-doped structures tend to exhibit stronger affinity toward soft ions through HSAB-driven binding.^51–53^ Similarly, defect-rich MQDs can facilitate redox-mediated electron transfer by introducing mid-gap states that serve as charge trapping or transfer sites. These structural features ultimately modulate exciton recombination dynamics, fluorescence lifetimes, and emission intensities that form the optical signal used for sensing.

Importantly, the mechanisms described here provide predictive guidelines for rational sensor design. By tailoring MQD surface chemistry and electronic structure, it becomes possible to bias the system toward specific interaction pathways that enhance selectivity and sensitivity toward target ions. In this way, mechanistic understanding transforms MQDs from passive fluorescent materials into engineered sensing platforms.^59,60^ The following section builds upon this framework by examining how these interaction principles translate into analytical sensing performance for environmentally and geochemically relevant ions.

## 4 Performance-driven applications of MQDs in earth-relevant ion sensing

The mechanistic principles discussed in Section 3 provide the essential foundation for understanding the analytical performance of MQD-based ion sensors. Electrostatic attraction, coordination binding, redox-driven electron transfer, and energy transfer processes are not merely theoretical constructs; they directly dictate key sensing parameters such as detection limit, selectivity, response time, dynamic range, and operational stability. For example, strong coordination affinity enhances selectivity toward specific ions, while favorable redox potential alignment promotes efficient charge transfer and amplified fluorescence modulation. Similarly, engineered defect states and heterostructured interfaces influence exciton recombination dynamics, thereby determining signal intensity and reversibility. The following case studies illustrate how deliberate nano-architectonic design and controlled interaction pathways translate into measurable analytical advantages in environmentally and geochemically relevant ion detection systems.

### 4.1 Cadmium ion detection *via* heterostructured QDs

The detection of cadmium ions (Cd^2+^) using MXene-based quantum dots is particularly effective when employing heterostructured composites. The combination of CsPbBr_3_ perovskite quantum dots with Ti_3_C_2_T_*x*_ MQDs creates a van der Waals heterojunction that enables pronounced photoluminescence (PL) quenching upon interaction with Cd^2+^ ions. The mechanism relies on charge transfer from the perovskite QDs to the MQDs, followed by rapid energy relaxation of hot carriers, which results in a significant reduction of steady-state PL intensity. Time-resolved studies further demonstrate that this energy transfer occurs on sub-nanosecond timescales, indicating efficient electron–hole separation.

The on–off–on PL response allows real-time, reversible monitoring of Cd^2+^, making the system highly suitable for environmental sensing.^75^ The combination of strong absorption in the visible range and fast electron transfer provides an intrinsic amplification mechanism, increasing sensitivity and selectivity. Importantly, the use of MXene QDs as electron acceptors not only facilitates quenching but also stabilizes the perovskite structure, mitigating photobleaching effects.

From a performance standpoint, the heterostructured QD system exhibits a detection limit that surpasses conventional fluorescent sensors while maintaining operational stability under varying pH and ionic strength conditions. This dual-functionality—detection and structural stabilization—illustrates the strategic advantage of MXene integration and highlights the potential for designing robust ion sensors for heavy metal contamination in aqueous environments.

**Fig. 4:**
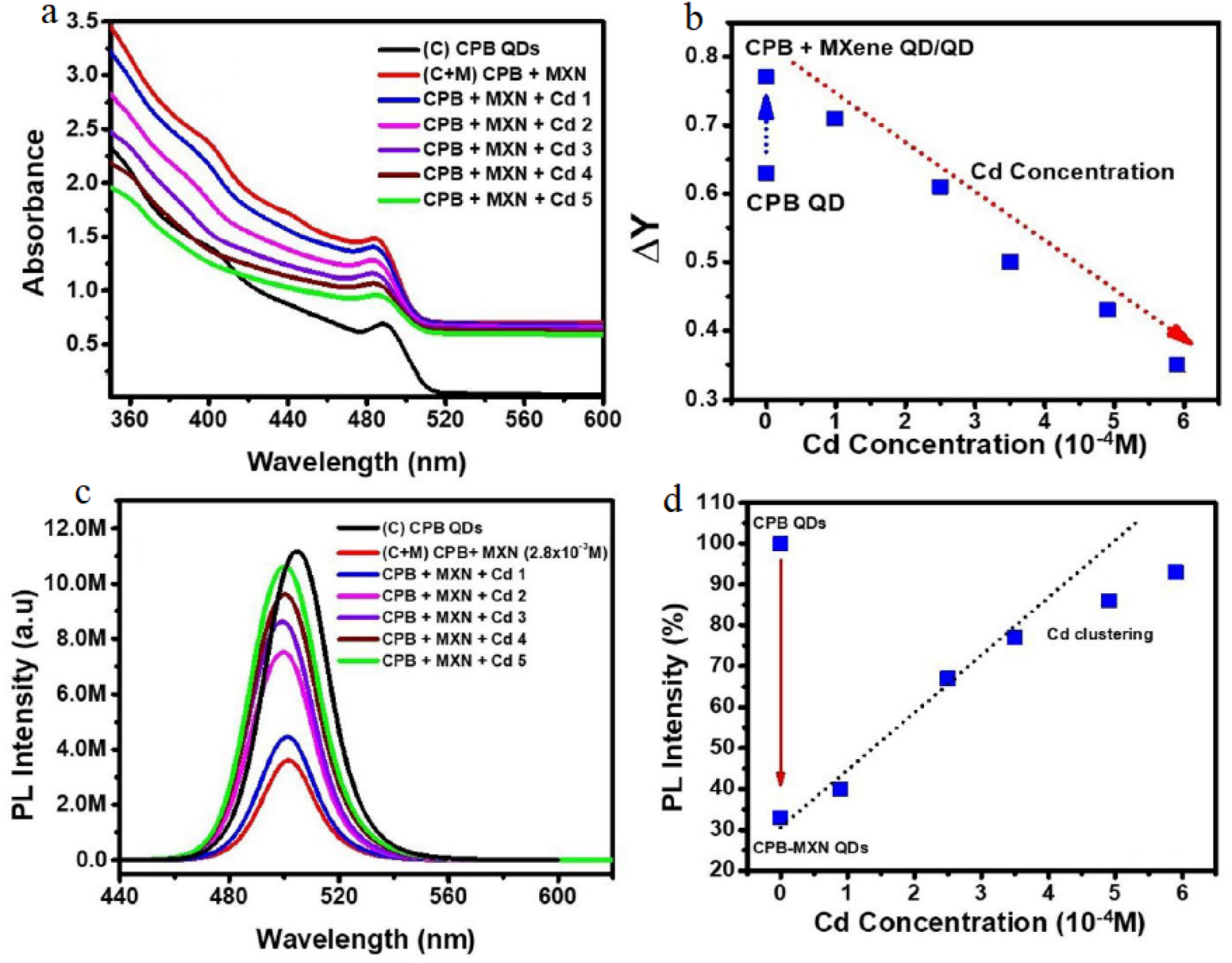
Optical response of CPB–MXN heterostructured QDs toward Cd^2+^ ions. (a) Absorption spectra of CPB QDs and CPB–MXN composites with increasing Cd^2+^ concentration. (b) Variation of effective absorbance (Δ*Y*) as a function of Cd^2+^ concentration. (c) Steady-state photoluminescence spectra showing Cd^2+^-induced emission recovery in the CPB–MXN system. (d) Relative PL enhancement *versus* Cd^2+^ concentration, demonstrating quantitative sensing performance. This figure has been reproduced from ref. 75 with permission from American Chemical Society, copyright 2020.

The spectroscopic trends presented in Fig. 4a and b suggest the formation of an electronically coupled CPB–MXN heterostructure; however, the interpretation of these features as definitive evidence of Cd^2+^-induced interfacial electronic modulation requires further substantiation. While the enhanced absorption beyond the CPB band edge in the composite can reasonably be attributed to the contribution of MXene quantum dots with narrower electronic gaps, distinguishing genuine interfacial electronic coupling from possible physical effects—such as light scattering or inner-filter effects—remains essential. Moreover, the observed decrease in effective absorbance (Δ*Y*) with increasing Cd^2+^ concentration is interpreted as perturbation of the heterojunction electronic structure, yet similar spectral variations could also arise from partial surface passivation, aggregation, or compositional instability of the perovskite QDs in the presence of metal ions. Therefore, complementary analyses (*e.g.*, XPS, electrochemical impedance spectroscopy, or comparative measurements with pristine CPB QDs exposed to Cd^2+^) would be necessary to directly confirm that the spectral shifts originate from changes in interfacial charge distribution rather than secondary physicochemical effects.

The photoluminescence behavior illustrated in Fig. 4c and d further supports the proposed sensing mechanism, but the mechanistic interpretation should be considered with caution. The recovery of fluorescence intensity after Cd^2+^ addition is attributed to partial suppression of non-radiative electron transfer from CPB to MXN; however, without time-resolved photoluminescence measurements, it remains difficult to clearly distinguish whether the process arises from modulation of carrier transfer dynamics, static complex formation, or surface-state passivation. In addition, although Fig. 4d indicates a near-linear relationship between PL intensity and Cd^2+^ concentration at lower levels, the absence of statistical indicators such as error bars or replicate measurements limits the quantitative reliability of the sensing calibration. The deviation from linearity at higher concentrations, attributed here to surface saturation or ion clustering, may also reflect reduced selectivity or competitive interactions at active sites. Consequently, further evaluation of interference from structurally similar ions and validation of detection limits under realistic aqueous conditions would strengthen the interpretation of the heterostructured CPB–MXN system as a robust and selective Cd^2+^ sensing platform.

### 4.2 Manganese(vii) detection and dual-function scavenging

Ti_3_C_2_ MQDs exhibit intrinsic reducibility that can be harnessed for both ultrasensitive detection and remediation of Mn(vii) ions. Hydrothermally prepared MQDs with surface functionalization display high dispersibility and fluorescence stability, enabling the development of a fluorescent probe with detection limits as low as 5.2 nM. The mechanism involves a redox reaction between Mn(vii) and the MXene surface, producing rapid quenching of fluorescence concomitant with removal of the toxic species. In comparison, carbon dots derived from the same material but lacking inherent reducibility exhibit a significantly higher detection limit (230 nM), emphasizing the unique electronic properties of MXene QDs.

The dual-function capability enables simultaneous monitoring and scavenging *in situ*, as demonstrated in plant leaf applications where Mn(vii) levels were quantitatively evaluated and reduced. This versatileity is particularly important for environmental applications, where sensors must not only report the presence of hazardous ions but also contribute to their mitigation. The synergy between redox activity and photoluminescence response creates a robust platform capable of functioning under a range of environmental conditions.^76^

Analytically, the combination of static quenching and inner filter effects enhances selectivity and limits interference from coexisting ions. The redox-driven fluorescence response also facilitates rapid detection kinetics, often within seconds. This establishes Ti_3_C_2_ MQDs as a versatile template for designing dual-functional sensors for transition metal oxoanions, reinforcing the concept that intrinsic surface chemistry of MXenes can be engineered to address both sensing and remediation challenges.

### 4.3 Chromium(vi) detection *via* nitrogen-doped MQDs

Nitrogen-doped Ti_3_C_2_ MQDs (N-MQDs) demonstrate selective and highly sensitive detection of Cr(vi) ions through a characteristic on–off–on fluorescent mechanism. The initial fluorescence quenching occurs due to static quenching and inner filter effects when Cr(vi) binds to the functionalized MQD surface. Subsequent fluorescence recovery is triggered by the redox reaction with ascorbic acid, restoring emissive properties. This dual-step response allows precise quantification of Cr(vi) with detection limits down to 12 nM and enables simultaneous monitoring of coexisting reductants. From a performance perspective, N-MQDs exhibit excellent excitation-dependent photoluminescence and photobleaching resistance, maintaining stability over a wide concentration range (0.1–500 µM).^77^

The functionalization with nitrogen enhances binding affinity to Cr(vi), while preserving optical robustness. Such features are critical for potential platforms for environmental monitoring where environmental matrices may introduce interference. The system demonstrates a fine balance between selectivity, sensitivity, and dynamic range, showing that subtle surface engineering of MQDs can dramatically enhance sensing performance. Furthermore, the reversible “on–off–on” response allows real-time monitoring and potential reusability, supporting the design of sustainable, high-performance chromium sensors. The approach illustrates a broader principle: careful doping and surface chemistry tuning can be leveraged to generate versatile MQDs tailored for specific oxyanion detection in aqueous and environmental contexts.

**Fig. 5:**
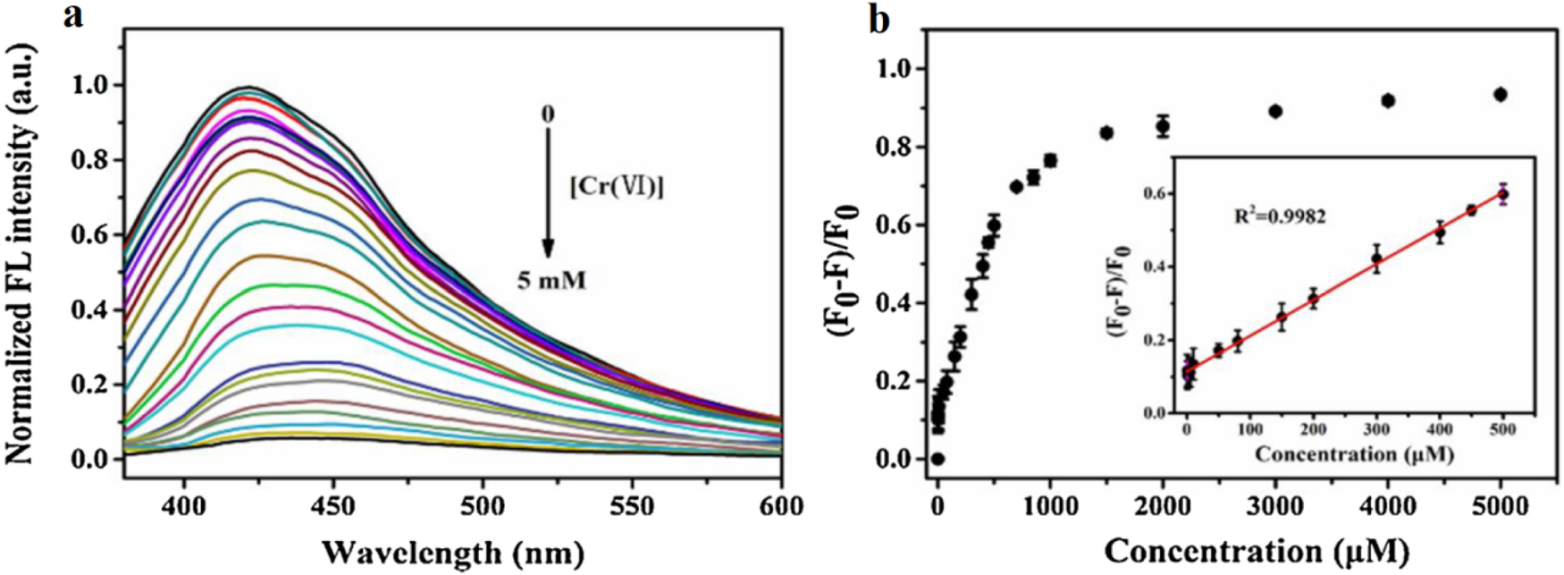
Redox-triggered fluorescence recovery of N–Ti_3_C_2_ MQDs for Cr(vi) detection. (a) Fluorescence spectra showing quenched emission by Cr(vi) and progressive restoration upon addition of AA. (b) Corresponding fluorescence recovery efficiency with linear calibration inset for AA quantification. This figure has been reproduced from ref. 77 with permission from Elsevier, copyright 2021.

Fig. 5 illustrates the redox-mediated sensing behavior of N-doped Ti_3_C_2_ MQDs toward Cr(vi); however, the mechanistic interpretation requires more rigorous validation. In Fig. 5a, the observed fluorescence quenching upon Cr(vi) addition is attributed to static quenching and inner-filter effects arising from surface binding at nitrogen-functionalized sites, yet the absence of supporting evidence—such as time-resolved photoluminescence measurements or absorption overlap analysis—makes it difficult to clearly distinguish between static quenching, dynamic quenching, or simple spectral filtering. Likewise, the fluorescence recovery after the introduction of ascorbic acid is interpreted as the reduction of Cr(vi) and restoration of the emissive MQD state, but additional confirmation of the redox transformation (*e.g.*, spectroscopic verification of Cr(vi) → Cr(iii)) would strengthen this claim. In Fig. 5b, the reported linear relationship between fluorescence recovery efficiency and AA concentration suggests promising quantitative capability; however, the reliability of this calibration would benefit from statistical validation, including replicate measurements, error bars, and interference studies with other reductants or metal ions commonly present in environmental matrices. Therefore, while the N-MQD system shows clear potential for reversible Cr(vi) sensing, further mechanistic clarification and robustness testing are necessary to fully substantiate its selectivity, sensitivity, and applicability in real environmental conditions.

### 4.4 Dichromate detection and rapid adsorption

Nitrogen and boron co-doped MXene quantum dots (NB-MQDs) embedded in functionalized pulp fiber papers provide a dual-function platform for Cr_2_O_7_^2−^ detection and removal. The combination of heteroatom doping and high surface area enhances both fluorescence response and adsorption kinetics. NB-MQDs@PP exhibits rapid recognition of Cr_2_O_7_^2−^ within 10 seconds, with a quenching efficiency of up to 99.9%, demonstrating an exceptionally high signal-to-noise ratio suitable for real-time environmental monitoring. The linear response range and low detection limits (3.8 nM for cyclic filtration) surpass conventional adsorbents, highlighting the role of MXene QDs in enhancing both analytical performance and removal efficiency.^78^

The mechanism is attributed to strong surface interactions facilitated by co-doping, which stabilizes electron transfer between the QDs and the oxyanion, while the porous substrate ensures rapid mass transport. Immersion and cyclic filtration methods were demonstrated as potential platforms for environmental monitoring, underscoring practical relevance. Adsorption capacity, reaching 162.4 mg g^−1^, also indicates high material efficiency, allowing simultaneous sensing and remediation, a combination rarely achieved in traditional inorganic adsorbents. NB-MQDs@PP exemplifies the integration of optical and chemical functionalities in a single platform. By engineering the surface chemistry of MXene QDs, both sensitivity and selectivity toward dichromate ions are maximized, while rapid kinetics ensures practical utility.

### 4.5 Arsenic sensing with nitrogen-doped MQDs

Bright yellow nitrogen-doped Ti_3_C_2_ MQDs (N-MQDs) provide an robust platform for selective As^3+^ detection. The MQDs exhibit a characteristic “on/off/on” fluorescence response: initial quenching occurs *via* static complex formation between As^3+^ and surface functional groups, followed by fluorescence recovery upon introduction of 2-amino-6-methoxybenzothiazole (MBTZ). The detection limit is as low as 30 nM, with a wide linear range, emphasizing the high sensitivity achievable through surface engineering. The yellow emission at 570 nm also facilitates deep-tissue penetration and reduces interference from background fluorescence in complex aqueous matrices. From a mechanistic perspective, the strong interaction between MBTZ and As^3+^ effectively detaches the ion from the MQD surface, restoring the emissive state. This dynamic response enables real-time monitoring and reusability, critical for environmental applications.^79^

Moreover, fabrication of solid-state sensors demonstrates practical on-site applicability, addressing limitations of solution-based sensing approaches. The performance-driven advantages of N-MQDs are twofold: high selectivity through functionalization and long-wavelength emission for operational versatility. These attributes indicate that careful design of heteroatom doping and surface functional groups allows precise control over ion-specific interactions, establishing a blueprint for robust arsenic detection platforms capable of combining optical sensitivity with practical deployment.

### 4.6 Mercury and chromium dual detection

Cr_2_C and potassium permanganate-functionalized Ti_3_C_2_ QDs provide a dual-ion detection strategy for Hg^2+^ and Cr^3+^. Cr_2_C MQDs show high selectivity for Hg^2+^ with detection limits of 30.7 nM, driven by strong affinity to surface functional groups. In parallel, Mn-QDs (KMnO_4_-modified Ti_3_C_2_ QDs) enable dual detection, leveraging electrostatic binding and surface trap modification to enhance photoluminescence in the presence of both Hg^2+^ and Cr^3+^. Dynamic quenching and defect passivation mechanisms contribute to precise signal modulation, resulting in low detection limits of 0.16 µM for Hg^2+^ and 0.80 µM for Cr^3+^, with high recovery rates (93–105%) in real water samples.^80,81^

The dual-sensing platform integrates selective adsorption, surface chemistry modulation, and luminescence enhancement, offering simultaneous detection without interference. Surface amino and oxygen functionalities on MQDs act as binding sites, while the functionalized QDs maintain colloidal stability and dispersibility, ensuring robust operation in complex environmental matrices. This approach demonstrates the potential of MXene QDs for multiplexed sensing applications, expanding their utility beyond single-ion detection.

By combining Cr_2_C and Mn-QD strategies, a versatile capable of addressing mercury and chromium contamination simultaneously. The results emphasize the importance of surface functionalization and heteroatom modification in tuning both selectivity and sensitivity, illustrating the adaptability of MXene QDs for multi-target environmental monitoring.

### 4.7 Emerging trends in versatile MQDs for integrated detection and remediation

Recent advances in MQDs have highlighted the development of versatile platforms that transcend conventional single-analyte sensing. The intrinsic tunable reducibility, surface functionalization, and photoluminescence of Ti_3_C_2_-based MQDs enable simultaneous detection and removal of metal ions, a feature that positions these materials as robust sensing platforms.^76^ For instance, MQDs can undergo redox reactions with highly oxidizing ions like Mn(vii), leading not only to ultrasensitive fluorescence quenching but also to *in situ* scavenging of the target ion in environmental matrices. This dual-functionality represents a shift from purely analytical applications toward integrated detection-remediation strategies.^78^

The incorporation of elemental doping and co-doping strategies has further extended the functional versatility of MQDs. Nitrogen-doped MQDs demonstrate exceptional stability, excitation-dependent photoluminescence, and on/off/on fluorescence response mechanisms, enabling selective detection of Cr(vi) and ascorbic acid through combined inner filter and redox effects.^77^ Similarly, boron and nitrogen co-doped MQDs embedded in paper-based substrates facilitate rapid adsorption and sensitive monitoring of dichromate ions, with detection limits reaching the nanomolar range, illustrating the potential, high-performance environmental sensing platforms.^78^

Beyond ion sensing, recent studies have focused on dual-mode and multi-signal MQD sensors, where fluorescence is coupled with either colorimetric, ratiometric, or peroxidase-like catalytic responses. Ti_3_C_2_ QDs exhibit an analyte-perturbed balance between reducibility and fluorescence for dual-mode detection of silver ions, optimizing selectivity and providing self-verified signal reliability. Ratiometric detection strategies, such as for hypochlorite, employ MQDs to achieve naked-eye visualization alongside quantitative fluorescent readouts, enhancing usability in complex field samples. Additionally, hybrid systems like Fe_3_O_4_@Ti_3_C_2_ MQDs exploit synergistic peroxidase-like activity for sensitive colorimetric determination of Cr(vi), highlighting the convergence of enzymatic mimicry and photoluminescence for versatile sensing.^78,80^

### 4.8 Benchmarking and limitations of MQD-based ion sensors

While MQDs have demonstrated remarkable potential in ion sensing, a critical comparison with other nanomaterial platforms is necessary to contextualize their performance and identify current limitations. Compared with carbon dots and graphene quantum dots, MQDs often exhibit stronger charge-transfer interactions due to their metallic conductivity and surface terminations (–O, –OH, –F), which can enhance fluorescence modulation and lower detection limits for certain heavy metal ions. In contrast, semiconductor quantum dots typically offer higher intrinsic photoluminescence quantum yields, while metal–organic frameworks (MOFs) provide exceptionally high surface area and tunable pore environments that can promote selective adsorption of specific ions.

From an analytical perspective, many MQD-based sensors report detection limits in the nanomolar range, comparable to or better than several carbon-based quantum dots, particularly when heteroatom doping or heterostructure formation is employed.^69–71^ However, selectivity remains a challenge in complex aqueous matrices where competing ions may interact with similar surface functional groups. Additionally, the surface chemistry of MQDs can vary significantly depending on synthesis conditions, which may lead to batch-to-batch variability in sensing performance.

Another limitation involves the stability of surface terminations under oxidative or high-ionic-strength environments, potentially affecting long-term sensor reliability. In comparison, MOF-based sensors sometimes offer higher structural stability and more predictable host–guest interactions. Furthermore, although MQDs enable rapid electron-transfer-driven sensing mechanisms, systematic benchmarking studies across different nanomaterials are still relatively limited in the literature.^73–75^ Overall, MQDs provide a unique balance of conductivity, tunable surface chemistry, and strong interfacial charge-transfer capability, but further research is required to improve selectivity, structural stability, and reproducibility to fully compete with other established nanomaterial sensing platforms.

Table 2 provides a comparative benchmarking of MQD-based ion sensors with other representative nanomaterial platforms widely used in optical and electrochemical sensing. The comparison highlights differences in detection limits, dominant sensing mechanisms, and selectivity characteristics. MQDs offer a distinctive combination of high electrical conductivity and chemically active surface terminations, enabling efficient charge-transfer-driven sensing, although challenges in stability and reproducibility remain.

**Table 2:** Benchmark comparison of MQD-based ion sensors with other nanomaterial sensing platforms

Material platform	Typical detection limit (LOD)	Dominant sensing mechanism	Selectivity characteristics	Key advantages	Major limitations
MXene quantum dots (MQDs)	∼0.5–50 nM	Interfacial charge transfer, fluorescence quenching/enhancement, surface complexation with terminal groups (–O, –OH, –F)	Moderate to high; strongly dependent on surface terminations and functionalization	High electrical conductivity, abundant active sites, rapid electron transfer kinetics, tunable surface chemistry	Surface oxidation, variability in termination chemistry, reproducibility issues between synthesis batches
Carbon dots (CDs)	∼5–500 nM	Fluorescence quenching (photoinduced electron transfer), surface coordination with oxygen/nitrogen groups	Moderate; often requires heteroatom doping or ligand modification	Simple synthesis, good aqueous stability, low toxicity	Lower conductivity and weaker electronic coupling with target ions
Graphene quantum dots (GQDs)	∼1–100 nM	π–π interactions, fluorescence modulation, surface adsorption and electron transfer	Moderate; improved through edge functionalization	High photostability, tunable edge chemistry, strong fluorescence stability	Limited density of specific binding sites without functionalization
Semiconductor quantum dots (*e.g.*, CdSe, CdTe)	∼0.01–10 nM	Fluorescence quenching/enhancement, Förster resonance energy transfer (FRET), exciton recombination modulation	High after ligand engineering or surface passivation	Very high photoluminescence quantum yield, strong optical response	Potential toxicity of heavy metals, complicated synthesis and surface modification
Metal–organic frameworks (MOFs)	∼0.01–5 nM	Host–guest interactions, fluorescence modulation, ion adsorption within porous framework	High due to tunable pore size and coordination sites	Extremely high surface area, structurally tunable recognition environments	Lower intrinsic electrical conductivity, sometimes limited stability in aqueous media

### 4.9 Emerging sensing principles and cross-ion trends in MQD-based sensors

Beyond individual case studies, MQD-based ion sensors exhibit several recurring sensing principles that provide unifying insight across different analytes. For most heavy metal ions such as Cd^2+^ and Hg^2+^, the dominant mechanism involves surface complexation with terminal groups (–O, –OH, –F or grafted ligands), leading to photoinduced electron transfer and efficient fluorescence quenching or enhancement. In contrast, strongly oxidizing species such as Cr(vi) and Mn(vii) frequently engage in redox-driven charge transfer, in which MQDs act as electron donors, perturbing exciton recombination pathways and modulating photoluminescence intensity or lifetime.

Cross-comparison of reported systems reveals that high-affinity coordination (*e.g.*, soft–soft interactions for Hg^2+^) generally yields lower detection limits and better selectivity, whereas ions with similar hard/soft character or coordination preferences tend to generate cross-sensitivity. Furthermore, MQD architectures that integrate heteroatom doping, core–shell structures, or hybridization with polymers and MOFs consistently show improved signal-to-noise ratios and reduced interference in complex matrices.^35,68,76^ Overall, these trends indicate that interfacial charge transfer, governed by ion-specific coordination chemistry and electronic coupling at the MQD surface, is the overarching sensing principle, with fluorescence modulation emerging as the most widely exploited readout mechanism across MQD-based ion-sensing platforms.

### 4.10 Critical assessment of mechanistic interpretation and practical reliability

Despite the rapid progress in MQD-based ion sensing, several conceptual and practical challenges remain that warrant careful consideration. A common limitation across many reported studies is the reliance on steady-state fluorescence measurements as the primary evidence for sensing mechanisms. Although changes in photoluminescence intensity provide a convenient analytical signal, they often do not uniquely identify the underlying process. Mechanisms such as static quenching, dynamic quenching, photoinduced electron transfer, inner-filter effects, and surface complexation can produce similar spectral signatures. Without complementary techniques—such as time-resolved photoluminescence, transient absorption spectroscopy, or electrochemical characterization—it is difficult to conclusively determine whether fluorescence modulation originates from genuine charge-transfer interactions or from secondary optical artifacts.

Another issue concerns the interpretation of selectivity in multicomponent environments. Many MQD sensors demonstrate excellent sensitivity toward a target ion under controlled laboratory conditions; however, the reported selectivity is frequently evaluated against a limited set of competing ions and often at equal concentrations that may not reflect realistic environmental matrices. In natural waters, the presence of multiple metal ions, organic ligands, and variable ionic strength can significantly alter surface interactions and binding equilibria.^74–76^ Consequently, fluorescence responses attributed to specific ion coordination may partially arise from competitive adsorption, surface passivation, or changes in local dielectric environment.

Reproducibility and material variability also represent important challenges. The surface chemistry of MQDs is highly dependent on synthesis parameters such as etching conditions, precursor composition, and post-treatment steps. Small variations in terminal groups (–O, –OH, –F) or defect density can substantially modify charge-transfer efficiency and fluorescence behavior. As a result, batch-to-batch variability may influence sensor calibration, detection limits, and long-term stability, complicating direct comparison between different studies.^78–80^

From an application perspective, another limitation is the insufficient validation of sensing performance in realistic environmental conditions. While many reports highlight nanomolar detection limits, fewer studies systematically evaluate long-term stability, photobleaching resistance, or performance under fluctuating pH, salinity, and oxidative conditions. Addressing these issues will require more standardized benchmarking protocols, integration of multiple spectroscopic techniques to confirm mechanisms, and broader testing in real water samples. Such efforts will be essential for translating MQD-based ion sensors from promising laboratory demonstrations into reliable tools for environmental monitoring and geochemical analysis.

## 5 Challenges and future perspectives: towards high-performance geochemical sensing platforms

The advancement of MQDs for geochemical ion sensing has opened unprecedented avenues for environmental monitoring and earth sciences. Despite significant progress demonstrated in recent years, there remain critical challenges that must be addressed to transition from laboratory-scale investigations to robust, and intelligent sensing platforms. One fundamental challenge lies in the reproducible synthesis of MQDs with uniform size, controlled surface functionality, and stable photoluminescence properties. Current hydrothermal, chemical, or top-down exfoliation approaches, although effective in generating fluorescent MQDs, often yield heterogeneity in particle size and surface chemistry. Such variability directly impacts sensitivity, selectivity, and detection limits, potentially complicating real-world deployment where environmental matrices are complex and dynamic.^38,75^ Precise synthetic control, possibly through automated or microfluidic-assisted protocols, will be critical for producing MQDs that are both highly functional and reproducible across batches.

In addition to these synthetic challenges, several fundamental limitations must be more explicitly acknowledged to provide a realistic outlook on the practical deployment of MQD-based sensing technologies. First, the potential toxicity of MXenes and their quantum dot derivatives remains insufficiently characterized, particularly regarding long-term biological and ecological impacts. This raises concerns for *in situ* environmental applications where direct release or accidental exposure is possible. Second, oxidation instability continues to be a critical barrier; MXene-derived MQDs are prone to surface oxidation under ambient conditions, which can alter their electronic structure, quench photoluminescence, and reduce sensing reliability over time.

Third, batch-to-batch reproducibility is still a major issue, as slight variations in precursor composition, etching efficiency, or storage conditions can lead to significant performance discrepancies across samples. This variability complicates sensor calibration and limits standardization for real-world use. Finally, scalability remains a practical constraint: although laboratory syntheses yield high-quality MQDs, translating these protocols into large-scale, cost-effective production systems without sacrificing optical quality or surface uniformity is still challenging. Collectively, these limitations highlight the need for systematic toxicity studies, robust anti-oxidation strategies, standardized synthesis workflows, and scalable manufacturing approaches to ensure that MQD-based sensing systems can evolve from promising laboratory tools into reliable components of future environmental monitoring technologies.

In addition to these synthetic challenges, several fundamental limitations must be more explicitly acknowledged to provide a realistic outlook on the practical deployment of MQD-based sensing technologies. First, the potential toxicity of MXenes and their quantum dot derivatives remains insufficiently characterized, particularly regarding long-term biological and ecological impacts. This raises concerns for *in situ* environmental applications where direct release or accidental exposure is possible. Second, oxidation instability continues to be a critical barrier; MXene-derived MQDs are prone to surface oxidation under ambient conditions, which can alter their electronic structure, quench photoluminescence, and reduce sensing reliability over time. Third, batch-to-batch reproducibility is still a major issue, as slight variations in precursor composition, etching efficiency, or storage conditions can lead to significant performance discrepancies across samples. This variability complicates sensor calibration and limits standardization for real-world use. Finally, scalability remains a practical constraint: although laboratory syntheses yield high-quality MQDs, translating these protocols into large-scale, cost-effective production systems without sacrificing optical quality or surface uniformity is still challenging. Collectively, these limitations highlight the need for systematic toxicity studies, robust anti-oxidation strategies, standardized synthesis workflows, and scalable manufacturing approaches to ensure that MQD-based sensing systems can evolve from promising laboratory tools into reliable components of future environmental monitoring technologies.

Another persistent limitation is long-term stability and resistance to environmental degradation. While doping strategies and surface functionalization can enhance photostability and redox robustness, prolonged exposure to variable pH, ionic strength, and competing ions in natural waters may lead to partial oxidation or aggregation, compromising sensing performance. Functionalized surface passivation techniques, including polymer encapsulation, covalent functionalization, or co-doping with multiple heteroatoms, represent promising strategies to mitigate degradation. Moreover, integrating MQDs into solid-state supports, such as polymeric membranes, paper-based substrates, or hydrogels, can enhance stability while enabling facile handling and on-site applications, as demonstrated with NB-MQDs@PP platforms for Cr_2_O_7_^2−^ detection and adsorption.^78^

The selectivity in complex matrices remains another major hurdle. Environmental and geological samples often contain a mixture of metal cations, oxyanions, and organic species that can interfere with photoluminescence or redox-based detection. While surface engineering, doping, and composite formation have been shown to improve selectivity—enabling, for example, on/off/on detection of As^3+^ or Cr(vi) ^77,79^ions—further refinement is required for multiplexed sensing. The design of orthogonal sensing motifs, where multiple recognition elements respond selectively to distinct ions without cross-interference, is an emerging avenue. This could involve combining MQDs with other nanomaterials such as perovskite QDs, carbon dots, or metal–organic frameworks, leveraging synergistic photophysical or chemical interactions to enable simultaneous multi-ion detection.^75^

From a performance perspective, real-time quantitative detection and data integration pose additional challenges. The dynamic quenching mechanisms and inner filter effects that underpin MQD fluorescence responses provide excellent sensitivity but may be influenced by fluctuating environmental parameters, such as temperature, light intensity, or matrix composition. Optimized calibration strategies, signal normalization, and ratiometric fluorescence approaches can improve quantitative reliability. Furthermore, coupling MQD sensors with high-performance electronics, wireless data acquisition, and machine learning algorithms offers the potential for automated monitoring and predictive analytics, paving the way for fully integrated geochemical sensing networks capable of continuous field deployment.

In terms of scalability and practical implementation, the integration of MQDs into device architectures is still in early stages. Solid-state sensors, paper-based platforms, and microfluidic devices have shown promise,^79,81^ but challenges remain in translating laboratory fabrication protocols to mass production without compromising performance. Cost-effective, high-throughput synthesis methods, combined with modular device designs, will be critical to ensure that MQD-based sensing technologies can be widely adopted for environmental and geochemical applications. In addition, strategies to regenerate or recycle MQD-based materials will be important for sustainability and long-term operation, particularly when targeting hazardous ions such as Hg^2+^ or Cr^3+^.^80^

Finally, the future of high-performance geochemical sensing platforms lies in the convergence of material innovation, device engineering, and data science. The unique combination of tunable photoluminescence, redox activity, and surface chemistry in MQDs positions them as ideal candidates for high-performance sensors capable of simultaneous detection, remediation, and predictive environmental monitoring. By addressing current challenges—synthetic control, stability, selectivity, real-time quantification, and device scalability—researchers can envision integrated systems where MQDs act as active sensing nodes within distributed networks, providing high-resolution spatiotemporal mapping of metal and oxyanion distributions in natural and industrial environments.^76,81^ Such platforms could revolutionize environmental monitoring, resource management, and geochemical risk assessment, moving beyond conventional laboratory assays toward autonomous, adaptive, and versatile sensing ecosystems.

In conclusion, while MXene QDs have already demonstrated remarkable performance in heavy metal and oxyanion sensing, their full potential will be realized only through multidisciplinary integration of sophisticated materials design, precision device engineering, and analytical intelligence. Addressing the outlined challenges will enable the creation of robust and intelligent geochemical sensing platforms, capable of transforming environmental monitoring and providing actionable insights for both natural and engineered systems. The path forward is therefore defined not only by materials innovation but also by strategic alignment with real-world deployment constraints, sustainability considerations, and the growing demand for high-performance, responsive, and versatile sensing technologies.

## Conflicts of interest

The authors declare that they have no known competing financial interests or personal relationships that could have appeared to influence the work reported in this paper.

## Data Availability

No primary research results, software or code have been included and no new data were generated or analysed as part of this review.
